# Procedural Outcomes of a Self-Expanding Transcatheter Heart Valve in Patients with Porcelain Aorta

**DOI:** 10.3390/jcm12030945

**Published:** 2023-01-26

**Authors:** Clemens Eckel, Johannes Blumenstein, Christina Grothusen, Vedat Tiyerili, Albrecht Elsässer, Guido Dohmen, Anna Zeckzer, Luise Gaede, Yeong-Hoon Choi, Efstratios I. Charitos, Christian W. Hamm, Won-Keun Kim, Helge Möllmann, Matthias Renker

**Affiliations:** 1 Department of Cardiology, St. Johannes Hospital, 44137 Dortmund, Germany; 2 Department of Cardiology, University of Oldenburg, 26129 Oldenburg, Germany; 3 Department of Cardiovascular Surgery, University Hospital of Schleswig-Holstein, Campus Kiel, 24118 Kiel, Germany; 4 Department of Cardiology, University of Bonn, 53113 Bonn, Germany; 5 Department of Cardiac Surgery, St. Johannes Hospital, 44137 Dortmund, Germany; 6 Department of Cardiology, University Hospital of Erlangen, 91054 Erlangen, Germany; 7 Department of Cardiac Surgery, Kerckhoff Heart Center, 61231 Bad Nauheim, Germany; 8 Department of Cardiology, Kerckhoff Heart Center, 61231 Bad Nauheim, Germany; 9 Department of Cardiology, Justus-Liebig University of Giessen, 35390 Giessen, Germany

**Keywords:** TAVI, self-expanding, THV, ACURATE, stroke, porcelain aorta

## Abstract

Background: Severe calcification of the ascending aorta increases the peri-operative risk for neurological complications in patients with severe aortic stenosis. Transcatheter aortic valve implantation (TAVI) seems to be an optimal treatment option in these patients. However, the impact of the extent of aortic calcification on procedural and neurological outcomes during TAVI is unclear. Methods: Data from 3010 patients with severe native aortic valve stenosis treated with ACURATE *neo/neo2* from May 2012 to July 2022 were evaluated and matched by 2-to-1 nearest-neighbor matching to identify one patient with porcelain aorta (PA) (*n* = 492) compared with two patients without PA (*n* = 984). PA was additionally subdivided into circumferential (classic PA) (*n* = 89; 3.0%) and non-circumferential (partial PA) (*n* = 403; 13.4%) calcification. We compared outcomes according to VARC-3 criteria among patients with and without PA and identified predictors for occurrence of stroke in the overall population. Results: Technical success (88.5% vs. 87.4%, *p* = 0.589) and device success at 30 days (82.3% vs. 81.5%, *p* = 0.755) after transcatheter ACURATE *neo/neo2* implantation according to VARC-3 definition was high and did not differ between non-calcified aortas or PA. The rate of in-hospital complications according to VARC-3-definitions was low in both groups. Rates of all stroke (3.2% (*n* = 31) vs. 2.6% (*n* = 13), *p* = 0.705) or transitory ischemic attacks (1.1% vs. 1.2%, *p* = 1.000) did not differ significantly. Thirty-day all-cause mortality did not differ (3.0% vs. 3.2%, RR 1.1; *p* = 0.775). Overall device migration/embolization (OR 5.0 [2.10;11.87]), severe bleeding (OR 1.79 [1.11;2.89]), and major structural cardiac complications (OR 3.37 [1.32;8.57]) were identified as independent predictors for in-hospital stroke in a multivariate analysis after implantation of ACURATE *neo/neo2*. Conclusion: A porcelain aorta does not increase the risk of neurological complications after transfemoral ACURATE *neo/neo2* implantation. Based on these findings, transfemoral ACURATE *neo/neo2* implantation is safe in these particularly vulnerable patients.

## 1. Introduction

Transcatheter aortic valve implantation (TAVI) has continued to advance in recent years in terms of experience, technique, and clinical application [1,2]. Peri-procedural risks of valve interventions can be assessed in advance using clinically established risk scores, such as the European System for Cardiac Operative Risk Evaluation Score (EuroSCORE II) or the Society of Thoracic Surgeons (STS) Score. Importantly, there are additional anatomically and technically relevant aspects that need to be considered but are not included in the abovementioned scores. In this context, the spectrum from advanced calcification of the ascending aorta to complete circular and unclampable porcelain aorta (PA) is generally considered to be an independent risk for and a relative contraindication to surgical aortic valve replacement [3]. From the beginning, TAVI was considered a valuable treatment alternative in patients with PA [4]. However, data on the impact of the presence and extent of aorta ascendens calcification on outcomes after transfemoral TAVI using new generation devices are scarce. Therefore, the aim of our analysis was to evaluate clinical outcomes of patients with or without PA treated by self-expanding ACURATE *neo/neo2*.

## 2. Materials and Methods

Consecutive patients with symptomatic severe native aortic stenosis who underwent transfemoral TAVI between May 2012 and July 2022 using the ACURATE *neo* (*n* = 2055) or ACURATE *neo2* (*n* = 955) prosthesis (Boston Scientific, Ecublens, Switzerland) were retrospectively included from two high-volume German centers (Kerckhoff Heart Center, Bad Nauheim, Germany; St. Johannes Hospital, Dortmund, Germany). The design and implantation technique of this transcatheter heart valve design have been described previously [5,6]. Baseline characteristics such as risk scores, comorbidities, MDCT (multidetector computed tomography), echocardiography, and cardiac catheterization data were recorded prospectively in a dedicated database as well as procedural data and complications from each participating center. Follow-up data were collected from recent medical reports, at outpatient visits, or by telephone interview. This study was conducted according to the Declaration of Helsinki. Due to its retrospective nature and anonymous data processing, ethical approval was waived by the respective local ethics committees.

### 2.1. Multidetector Computed Tomography

**Figure 1:**
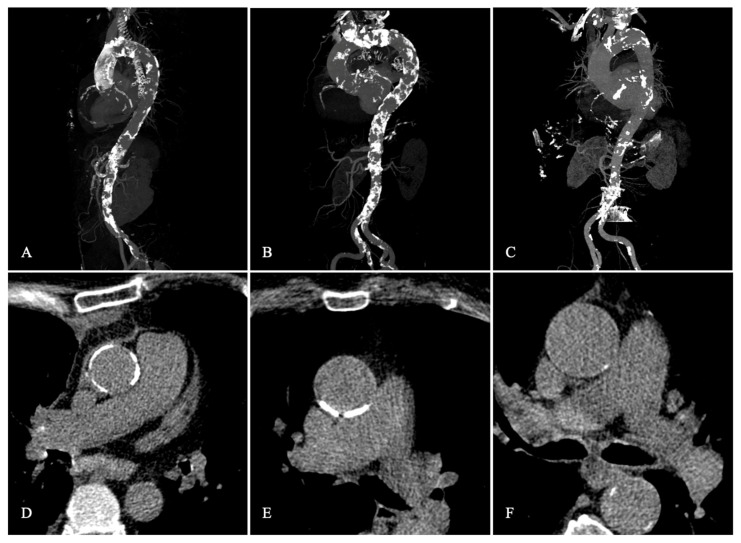
MDCT classification of the extent of ascending aorta atherosclerotic burden. Maximum intensity projection (MIP) of the aorto-iliac arteries demonstrating circular, near-confluent calcification of the ascending aorta (**A**); partial, non-confluent calcification (**B**); and absence of relevant atherosclerotic lesions of the ascending aorta (**C**). Axial views of the aorta at the level of the pulmonary trunk showing circular (**D**) and non-circular calcification (**E**) as well as no or minor calcification (**F**). Calcifications of the aortic arch and the descending aorta were not considered in the present analysis.

MDCT was performed using a 64-slice or a 192-slice dual-source scanner (Somatom Definition or Somatom Force, Siemens Healthcare, Forchheim, Germany) as previously described [7]. A dedicated software was used (3mensio, Pie Medical, Bilthoven, The Netherlands) for the analysis of MDCT datasets. The above standard measurements (aortic root dimensions), the cover index [CI = 100 × (prosthesis diameter − perimeter − derived annulus diameter)/prosthesis diameter (%)], and the relationship between the sinotubular junction (STJ) and the perimeter-derived annulus was calculated as STJ-annulus index [=100 × (STJ − perimeter − derived annulus)/STJ (%)]. We used the Agatston method using non-contrast-enhanced MDCT scans for measurement of the aortic valve calcium score (AVCS) [8]. We calculated calcium density (Ca-density) as AVCS/annular area (AU/cm^2^) [9]. The presence of eccentric aortic valve (AV) calcification and relevant left ventricular outflow tract (LVOT) calcification was determined by visual estimation of the aortic valve in short axis views and maximum intensity projections as previously described [10]. The atherosclerotic burden of the ascending aorta was assessed visually using both non-contrast axial CT sequences and maximum intensity projection (MiP) reconstructions (Figure 1). PA was assessed as either circular (not clampable) or non-circular (clampable) according to the VARC criteria [11].

### 2.2. Outcome Analysis

The primary outcome measure was in-hospital mortality and occurrence of in-hospital stroke. Secondary outcome measures were 30-day all-cause mortality, 30-day stroke, technical success, device success at 30 days, and the early safety combined endpoint at 30 days according to the recent VARC-3 document [12].

### 2.3. Definition of Porcelain Aorta

Various approaches have been used in the past to define the phenomenon of PA. As with the VARC-2 criteria, the definition is usually based on “severe circumferential calcification or severe atheromatous plaques of the entire ascending aorta extending to the arch” [9], see Figure 1. The surgical perspective focuses on the possibility of cross-clamping of the ascending aorta [11]. From an interventional perspective, it remains unclear whether a retrograde manipulation of the calcium deposition with the valve and/or delivery catheter system promotes events such as stroke, embolization, or rupture of the aorta. Accordingly, no definition or classification exists yet that would provide a neurological injury risk stratification for TAVI procedures in patients with PA.

### 2.4. Statistical Analysis

Statistical analyses were conducted using dedicated software (R version 4.2.1 (2021) R Foundation for Statistical Computing, Vienna, Austria). The population was divided in two main subsets according to a calcified aorta, including full PA (*n* = 492) or non-PA (*n* = 2518). Patients with PA were subdivided into circular (*n* = 89) and non-circular (*n* = 403) calcification (Figure 1). Continuous data are presented as median and interquartile range (IQR). Comparison of groups was accomplished using the Fisher’s two-tailed exact test and the chi-square test or Mann–Whitney U test as indicated. To reduce the influence of potential confounders on patient outcomes and the effects of potential selection bias on endpoints when comparing PA with non-PA, propensity matching was performed using R Studio (matchit package). A 2-to-1 nearest-neighbor matching was used to identify a control case without PA (*n* = 984) for each patient with PA (*n* = 492). In addition, a subanalysis of the 2-to-1 nearest neighbor matched collective of circulating versus non-PA (*n* = 89 vs. *n* = 178) was performed (see Supplemental Table S4). Clinical history, CHA_2_DS_2_VASc score characteristics [13], and MDCT characteristics with known effects or significant (*p* < 0.05) univariate differences between the two groups were included in the matching algorithm (see Supplemental Table S4). Univariable logistic regression was used to determine predictors of stroke. All variables with *p*-values < 0.1 in the univariate analysis were included in the multivariable analysis. For all analyses, a two-sided *p*-value < 0.05 was considered significant.

## 3. Results

### 3.1. Baseline Data

The mean age was 82.0 years and 61.8% were female (see Table 1). After 2-to-1 nearest-neighbor-matching, there were no differences with respect to baseline patient characteristics, including comorbidities, regardless of classification as non-, partial PA, or circular PA (Table 2). See Supplemental Table S1 for baseline characteristics of matched population for classic PA.

### 3.2. Procedural Data and Outcomes

Table 3 provides procedural characteristics in the matched population (Supplemental Tables S2 and S3 for entire and circumferential population). A cerebral protection device was similarly restricted to selected cases of patients without and with PA (1.5% vs. 2.0%, *p* = 0.618, respectively). Pre- and post-dilatation were performed in 84.3% and 30.9%, respectively, without differences between the groups. Periprocedural complications according to VARC-3 criteria were comparable in both groups. Technical success was high in both groups (88.5 vs. 87.4%, *p* = 0.589). Technical failure was mainly driven by moderate PVL and intervention or surgery due to vascular complications (Supplemental Table S5). Early safety defined by VARC-3 at 30 days was equal (47.0 vs. 49.2%, *p* = 0.450), mainly driven by severe bleeding (20.5% vs. 23.8%, *p* = 0.173) and need for pacemaker implantation (10.1% vs. 12.0%, *p* = 0.306). Major cardiac structural complications were rare (1.4% vs. 0.8%, *p* = 0.450). Occurrence of overt central nervous system (CNS) injury (all stroke) was comparable between the groups (3.2% vs. 2.6%, *p* = 0.705) as well as neurologic dysfunction without CNS injury (TIA) was (1.1% vs. 1.2%, *p* = 1.000). There was no difference regarding stroke severity (fatal stroke vs. stroke with disability vs. stroke without disability) between patients without and with porcelain aorta (18.2% vs. 19.4%; 36.4% vs. 35.5%; 45.5% vs. 45.2%, *p* = 1.000, respectively). Excluding patients with non-disabling stroke or TIA, only 1.6% (*n* = 24) suffered from fatal or disabling stroke.

### 3.3. Predictors for Stroke

Table 4 shows uni- and multivariable logistic regression model for the occurrence of in-hospital stroke in the entire population. In the overall cohort, migration/embolization, major cardiac structural complications, and severe bleeding were independent predictors of in-hospital stroke. Atherosclerotic burden of the aorta (partial, circular, or both) did not serve as a predictor of the in-hospital occurrence of a stroke.

### 3.4. Outcome Analysis up to 30 Days

**Figure 2:**
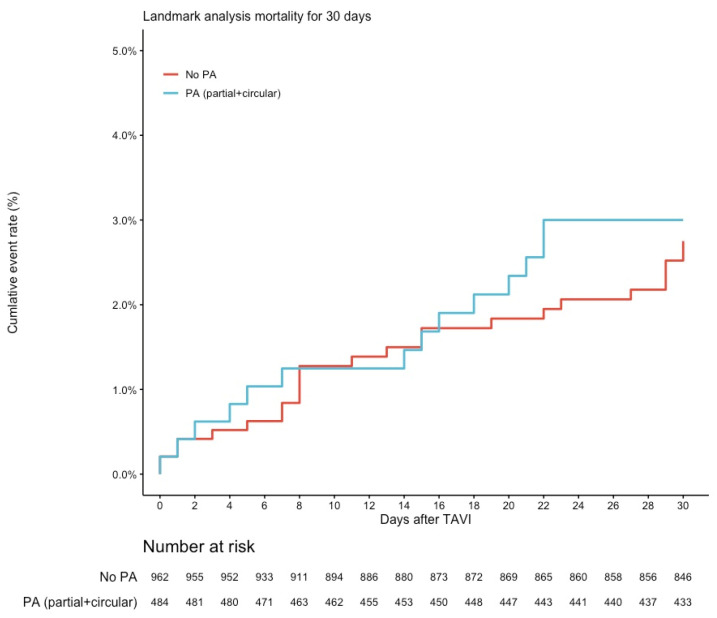
Kaplan–Meier Curve for mortality up to 30 days. Lost to follow-up at 30 days: *n* = 30 (2.0%). Abbreviation: PA, porcelain aorta.

**Figure 3:**
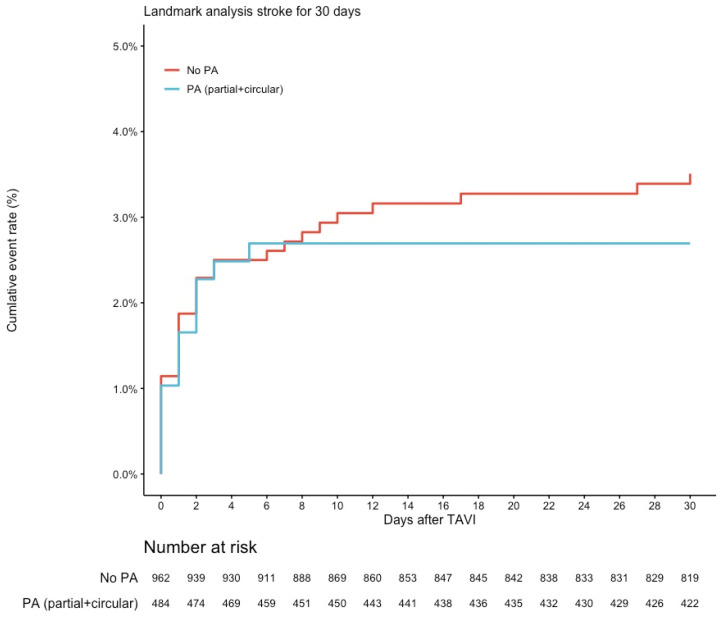
Kaplan–Meier Curve for stroke up to 30 days. Lost to follow-up at 30 days: *n* = 30 (2.0%). Abbreviation: PA, porcelain aorta.

There were no significant differences regarding 30-day mortality (2.8% vs. 3.0%, RR 1.10, 95% CI 0.57–2.12, *p* = 0.775) in the matched populations (Figure 2). No differences regarding the rate of stroke could be found intra-hospital or after a follow-up of 30 days (3.5% vs. 2.7%, RR 0.78, 95% CI 0.41–1.48, *p* = 0.447) (Figure 3).

## 4. Discussion

In addition to the continuous expansion of the TAVI spectrum including low-risk patients, there are concomitant diseases or anatomical aspects such as severe calcification of the ascending aorta that make surgical repair more complex regardless of the calculated risk scores. The occurrence of partial or circular PA varies in studies between 7.8–14.8% [14]. There are limited data on the use of newer generations of self-expanding valves in concomitant PA. Our main findings are: (1) ACURATE *neo/neo2* S showed favorable procedural outcomes even in patients with circular PA. (2) There was no significant difference in the rate or severity of stroke up to 30 days. (3) There was no significant difference in 30-day mortality. (4) Overall, independent predictors for stroke were device migration/embolization, major cardiac structural complications, and severe bleeding. (5) Atherosclerotic burden of the aorta (partial, circular, or both) could not be proven as a predictor of in-hospital stroke.

### 4.1. Procedural and In-Hospital Outcome

Few comparative data are available for SAVR vs. TAVI in PA. A small number of studies demonstrated reduced perioperative mortality and shorter ICU and in-hospital length of stay after TAVI [15]. The most recent systematic outcome studies in patients with severe native aortic valve stenosis and PA analyzed only small subsets with 147 [14], 114 [16], and 36 patients with PA [17]. The authors found a higher rate of myocardial ischemia [14] and periprocedural stroke [14,16] in patients with PA compared to patients without. Two study-groups reported a direct correlation between the general extent of calcification (per cm^2^) of the aorta and mortality at one year and ascribed this observation to an increased afterload due to increased vascular stiffness [18,19,20]. The present analysis studied a representative number of patients and could not demonstrate any procedural differences or differences in mortality up to 30 days between patients with and without PA.

### 4.2. Stroke

Stroke prevention is crucial in surgical procedure preparation. In general, predictors of stroke after transcatheter intervention include previous stroke/transient ischemic attack, smoking, mechanical devices, age, renal function, BSA, and previous valvular interventions [17]. Stroke is more common after balloon expandable TAVI [21] and post dilation [22]. Most recent studies favored the transfemoral approach over alternative access routes regarding stroke [16,17,23]. Data from the PARTNER 2 trial revealed (any) stroke rates at 30 days of 4.2% (2.3% disabling stroke) with transfemoral and 9.8% (6.0% disabling stroke) with transthoracic access route [24]. A recent study in 2600 patients (including 50.2% self-expanding devices) emphasized the role of PA as an independent predictor for stroke 30 days after transcatheter intervention [16] even when adjusting for known confounders. In contrast to this, Pascual et al. were not able to demonstrate a similar effect in a smaller population (*n* = 36/449) treated with CoreValve [25]. In contrast, the present analysis is based on a representative propensity-matched patient population that underwent TAVI using the supra-annular self-expanding ACURATE *neo/neo2* and excluded any differences between patients with and without PA. At a first glance, stroke rate seems to be high. However, it has to be taken into account the definition of stroke was made according to the latest VARC 3 criteria as any overt central nervous system injury. Excluding patients with non-disabling (minor) stroke or TIA, only 1.6% suffered from clinically relevant stroke. The rate of disabling and fatal stroke did not differ between the groups. The routine use of cerebral protection devices failed to demonstrate a significant effect on the incidence of all periprocedural strokes in transfemoral transcatheter treatment of native aortic valve stenosis [26]. Especially in patients with severe calcification of the aorta or/and the aortic arch, manipulation during positioning of a cerebral protection device might also be counterproductive. However, larger trials are needed to clearly answer this important topic. At this timepoint, protection devices could be considered as a case-by-case decision in patients at higher risk. In this study, the number of procedures with protection devices (*n* = 73; 2.43%) was too small to assess an effect in this particular subset. Due to such low event rates after TAVI, only large studies have the power to distinguish dedicated subgroups that benefit from protection devices. The presentation of severe bleeding as a predictor of stroke can be presumably explained by hemodynamic effects in terms of CNS injury in hemorrhagic shock as well as the delayed reversal of antithrombotic drugs.

### 4.3. Limitations

The present analysis is limited by its retrospective, non-randomized nature and the small number of patients. Furthermore, a clear, generally valid definition of PA is lacking and due to low incidence, and patients were included over a long period of time, which may have led to bias due to different procedural approaches (e.g., changes in pre/post dilatation strategies, single femoral access, and radial access for pigtail catheter) and learning curve effects. Imaging data were not analyzed by a core laboratory, and there was no adverse event monitoring. Atherosclerotic burden in ascending aorta, LVOT calcification, and eccentric AV calcification were graded visually without further quantification. A bias due to a different data acquisition of the MDCT data (64 vs. 192 slice) cannot be excluded.

## 5. Conclusions

Transfemoral TAVI using the ACURATE *neo/neo2* prosthesis is safe and feasible in patients with severely calcified or even unclampable porcelain aorta. There are no observable differences with respect to intrahospital complications according to VARC-3 criteria, including stroke or death. The atherosclerotic burden of the aorta (partial or circular) could not identified as a predictor for periprocedural or in-hospital stroke.

## Figures and Tables

**Table 1:** Baseline characteristics of the entire population.

Variable	Non PA	Partial PA	Circular PA	*p*-Value
	*n* = 2518	*n* = 403	*n* = 89	
**Demographic and clinical data**				
Age, years	82.0 [79.0;85.0]	81.0 [77.0;84.5]	82.0 [79.0;85.8]	0.003
Female gender, %	1558 (61.9%)	248 (61.5%)	53 (59.6%)	0.902
BMI, kg/m^2^	26.7 [23.9;30.4]	26.6 [24.1;30.3]	25.8 [23.7;30.1]	0.839
EuroSCORE II, %	3.3 [2.2;5.2]	3.6 [2.4;5.8]	3.7 [2.3;5.2]	0.051
eGFR, mL/min/1.73 m^2^	60.0 [44.0;79.0]	57.0 [40.5;76.0]	55.0 [43.0;73.0]	0.017
Peripheral artery disease	307 (12.2%)	58 (14.4%)	12 (13.5%)	0.447
Prior stroke	313 (12.4%)	50 (12.4%)	12 (13.5%)	0.957
Atrial fibrillation	976 (38.8%)	154 (38.2%)	35 (39.3%)	0.971
Coronary artery disease	1568 (62.3%)	273 (67.7%)	58 (65.2%)	0.099
Prior coronary intervention	912 (36.2%)	155 (38.5%)	34 (38.2%)	0.651
**Echocardiographic data**				
LV ejection fraction, %	63.0 [55.0;65.0]	60.0 [53.5;65.0]	60.0 [52.0;65.0]	0.057
Mean gradient, mmHg	41.0 [32.0;50.0]	41.0 [32.0;51.0]	41.5 [34.8;50.0]	0.954
AVA, cm^2^	0.7 [0.6;0.8]	0.8 [0.6;0.9]	0.7 [0.6;0.8]	0.010
**Electrocardiographic data**				
Right bundle branch block	234 (9.3%)	40 (10.0%)	13 (14.6%)	0.241
Left bundle branch block	225 (9.0%)	33 (8.2%)	8 (9.0%)	0.892
Atrioventricular block	441 (17.6%)	71 (17.8%)	13 (14.6%)	0.761
**MDCT data**				
Annular area, cm^2^	3.6 [3.5;4.2]	4.0 [3.5;4.7]	3.7 [3.5;4.5]	<0.001
Annulus diameter, mm	24.0 [22.8;25.2]	24.0 [22.7;25.3]	23.9 [22.5;25.2]	0.764
LVOT, mm	23.2 [21.5;24.9]	23.5 [21.7;25.0]	23.5 [21.6;24.8]	0.115
STJ, mm	27.8 [26.0;29.8]	28.1 [26.3;30.1]	28.0 [26.5;29.9]	0.232
Aortic valve calcification, AU	2173 [1415;3062]	2247 [1474;3305]	2488 [1585;3460]	0.077
Calcium density, AU/cm^2^	576 [378;807]	563 [398;791]	644 [395;918]	0.569
Calcification in LVOT	188 (7.5%)	43 (10.7%)	9 (10.1%)	0.066
Eccentric calcification	326 (13.0%)	34 (8.5%)	10 (11.2%)	0.037

Abbreviation: BMI, body mass index; eGFR, glomerular filtration rate; LV, left ventricle; AVA, aortic valve area; LVOT, left ventricular outflow tract; STJ, sinotubular junction.

**Table 2:** Baseline characteristics for matched population.

Variable	Non PA (2:1)	PA	*p*-Value
	*n* = 984	*n* = 492	
**Demographic and clinical data**			
Age, years	81.9 [78.3;85.0]	81.5 [77.4;85.0]	0.264
Female gender, %	596 (60.6%)	301 (61.2%)	0.865
BMI, kg/m^2^	26.8 [23.9;30.8]	26.4 [24.1;30.2]	0.366
EuroSCORE II, %	3.6 [2.3;5.7]	3.6 [2.3;5.6]	0.728
eGFR, mL/min/1.73 m^2^	57.0 [42.0;74.0]	57.0 [41.0;75.2]	0.907
Peripheral artery disease	137 (13.9%)	70 (14.2%)	0.937
Prior stroke	126 (12.8%)	62 (12.6%)	0.978
Atrial fibrillation	382 (38.8%)	189 (38.4%)	0.925
Coronary artery disease	665 (67.6%)	331 (67.3%)	0.953
Prior coronary intervention	392 (39.8%)	189 (38.4%)	0.638
**Echocardiographic data**			
LV ejection fraction, %	61.0 [51.8;65.0]	60.0 [53.0;65.0]	0.674
Mean gradient, mmHg	42.0 [32.0;50.0]	41.0 [32.0;50.0]	0.708
AVA, cm^2^	0.7 [0.6;0.9]	0.7 [0.6;0.9]	0.189
**Electrocardiographic data**			
Right bundle branch block	100 (10.2%)	53 (10.8%)	0.786
Left bundle branch block	75 (7.6%)	41 (8.3%)	0.707
Atrioventricular block	165 (16.9%)	84 (17.2%)	0.941
**MDCT data**			
Annular area, cm^2^	3.8 [3.5;4.6]	3.9 [3.5;4.6]	0.660
Annulus diameter, mm	24.2 [22.9;25.4]	23.9 [22.6;25.3]	0.100
LVOT, mm	23.6 [21.9;25.4]	23.5 [21.7;25.0]	0.273
STJ, mm	28.0 [26.2;29.9]	28.1 [26.3;30.1]	0.925
Aortic valve calcification, AU	2306 [1492;3250]	2286 [1504;3361]	0.712
Calcium density, AU/cm^2^	585 [377;807]	571 [396;810]	0.909
Calcification in LVOT	100 (10.2%)	52 (10.6%)	0.880
Eccentric calcification	90 (9.1%)	45 (9.1%)	1.000

Abbreviations: BMI, body mass index; eGFR, estimated glomerular filtration rate; LV, left ventricle; AVA, aortic valve area; LVOT, left ventricular outflow tract; STJ, sinotubular junction.

**Table 3:** Procedural outcomes and complications (matched population).

Variable	Non PA (2:1)	PA	*p*-Value
	*n* = 984	*n* = 492	
**Procedural parameter**			
Procedural duration, min	48.00 [38.00;60.00]	50.00 [40.00;66.00]	0.078
Contrast agent, mL	97.00 [65.00;120.00]	95.00 [70.00;120.00]	0.866
Pre-dilatation, %	828 (84.15%)	416 (84.55%)	0.899
Post-dilatation, %	293 (30.14%)	158 (32.24%)	0.447
Protection device, %	15 (1.52%)	10 (2.03%)	0.618
Depth NCC, mm	6.00 [4.00;6.60]	6.00 [4.00;7.00]	0.303
Depth LCC, mm	6.00 [4.50;7.00]	6.00 [5.00;7.00]	0.142
Cover index (annulus)	4.46 [2.57;6.67]	4.58 [2.60;6.56]	0.649
**Echocardiographic outcome**			
LV ejection fraction, %	64.00 [55.00;65.00]	63.00 [55.00;65.00]	0.816
Mean gradient, mmHg	8.00 [6.00;11.00]	8.00 [6.00;11.00]	0.303
AVA, cm^2^	1.80 [1.50;2.10]	1.80 [1.50;2.02]	0.591
iAVA, cm^2^/m^2^	0.96 [0.82;1.12]	0.94 [0.84;1.12]	0.877
**Procedural and clinical outcome**			
Technical success	871 (88.52%)	430 (87.40%)	0.589
Device success at 30 days	810 (82.32%)	401 (81.50%)	0.755
Early safety at 30 days	462 (46.95%)	242 (49.19%)	0.450
In-hospital death	23 (2.34%)	11 (2.24%)	1.000
Periprocedural death (in-hospital and up to 30 days)	30 (3.05%)	16 (3.25%)	0.958
Relevant PVL (>mild/trace)	35 (3.56%)	20 (4.07%)	0.739
More than mild PPM	25 (3.46%)	17 (4.91%)	0.328
Conversion to sternotomy	7 (0.71%)	2 (0.41%)	0.726
Multiple valves (ViV)	12 (1.22%)	5 (1.02%)	0.931
Device migration/embolization	19 (1.93%)	8 (1.63%)	0.837
Major vascular complication	70 (7.11%)	45 (9.15%)	0.204
Bleeding (type 2–4)	202 (20.53%)	117 (23.78%)	0.173
Major cardiac structural complication	14 (1.42%)	4 (0.81%)	0.450
All stroke	31 (3.15%)	13 (2.64%)	0.705
Neurologic dysfunction without CNS injury (TIA)	11 (1.12%)	6 (1.22%)	1.000
AKI (type 2–4)	41 (4.17%)	15 (3.05%)	0.360
New permanent pacemaker ^1^	89 (10.14%)	52 (12.01%)	0.306

Abbreviations: PA, porcelain aorta; THV, transcatheter heart valve; LCC, left coronary cusp; NCC, non-coronary cusp; LV, left ventricle; AVA, aortic valve area; iAVA, indexed aortic valve area; PVL, paravalvular leak; CNS, central nervous system; ppm, prosthesis-patient mismatch; AKI, acute kidney injury; TIA, transitory ischemic attack. ^1^ Excluded patients with pacemaker at baseline (*n* = 165).

**Table 4:** Predictors for stroke (in-hospital).

	Univariate	*p*-Value	Multivariate	*p*-Value
**Predictors**				
Age	1.02 (0.98,1.06)	0.412		
Gender (male)	0.7 (0.45,1.1)	0.111		
CAD	0.83 (0.54,1.26)	0.381		
LV ejection fraction	0.99 (0.98,1.01)	0.641		
Annulus area	0.34 (0.1,1.1)	0.071	0.41 (0.13,1.33)	0.138
Cover index, annulus	0.99 (0.92,1.07)	0.830		
BMI	1.00(0.98,1.02)	0.996		
EuroSCORE II	1.00 (0.99,1.02)	0.559		
Depth LCC	0.93 (0.85,1.02)	0.152		
Depth NCC	0.93 (0.86,1.02)	0.129		
LVOT calcification	1.27 (0.63,2.55)	0.521		
Aortic valve calcification	1.00 (0.99,1.00)	0.709		
Circular PA	1.51 (0.54,4.22)	0.454		
Circular or partial PA	0.84 (0.46,1.52)	0.550		
Pre dilatation	0.95 (0.57,1.57)	0.831		
Post dilatation	1.33 (0.87,2.04)	0.193		
Protection device	0.43 (0.06;3.15)	0.343		
**Migration/embolization**	6.41 (2.78,14.79)	<0.001	5.00 (2.10,11.87)	<0.001
**Major cardiac structural complication**	4.89 (2.02,11.83)	0.003	3.37 (1.32,8.57)	0.011
Prior pacemaker	0.35 (0.13,0.95)	0.016	0.40 (0.14,1.10)	0.075
**Severe bleeding**	2.29 (1.47,3.57)	<0.001	1.79 (1.11,2.89)	0.017
Prior atrial fibrillation	0.68 (0.44,1.07)	0.092	0.76 (0.48,1.20)	0.235

Abbreviations: CAD, coronary artery disease; LCC, left coronary cusp; NCC, non-coronary cusp; LVOT, left ventricular outflow tract; LV, left ventricular.

## Data Availability

Data is contained within the article.

## Supplementary Materials

The following supporting information can be downloaded at: https://www.mdpi.com/article/10.3390/jcm12030945/s1, Table S1: Baseline characteristics (matched population for classic PA); Table S2: Procedural outcomes and complications (matched population for classic PA); Table S3: Procedural outcomes and complications (entire population); Table S4: Matching Algorithm; Table S5: Technical Failure.

## Abbreviations

AVCS	Aortic valve calcium score
CI	Cover index
LVOT	Left ventricular outflow tract
MDCT	Multidetector computed tomography
PA	Porcelain aorta
PVL	Paravalvular leakage
STJ	Sinotubular junction
TAVI	Transcatheter aortic valve implantation
THV	Transcatheter heart valve
VARC	Valve academic research consortium
