# Supplementary material for: Procedural Outcomes of a Self-Expanding Transcatheter Heart Valve in Patients with Porcelain Aorta
Source: J Clin Med. 2023 Jan 26;12(3):945. doi: 10.3390/jcm12030945 (PMC9917710; doi:10.3390/jcm12030945)
Supplement: Supplementary file 1 [file jcm-12-00945-s001.zip › jcm-2134431-supplementary.pdf]

Table S1. Baseline characteristics (matched population for classic PA).

Variable	**Non PA**<br>**n=178**	**Circular PA**<br>**n=89**	**p Value**
**Demographic and clinical data**			
Age, years	82.2 [79.0;85.2]	82.0 [79.0;85.8]	0.952
Female gender	125 (70.2%)	53 (59.6%)	0.108
BMI, kg/m^2	26.8 [24.1;29.6]	25.8 [23.7;30.1]	0.600
EuroSCORE II, %	3.5 [2.3;5.2]	3.7 [2.3;5.2]	0.938
eGFR, ml/min/1,73 m^2	51.0 [34.2;71.0]	55.0 [43.0;73.0]	0.212
Peripheral artery disease	22 (12.4%)	12 (13.5%)	0.948
Prior stroke	27 (15.2%)	12 (13.5%)	0.854
Atrial fibrillation	76 (42.7%)	35 (39.3%)	0.693
Coronary artery disease	118 (66.3%)	58 (65.2%)	0.964
Prior coronary intervention	89 (50.0%)	34 (38.2%)	0.090
**Echocardiographic data**			
LV Ejection fraction, %	60.5 [52.0;65.0]	60.0 [52.0;65.0]	0.636
Mean gradient, mmHg	41.0 [31.2;49.8]	41.5 [34.8;50.0]	0.814
AVA, cm^2	0.7 [0.6;0.9]	0.7 [0.6;0.8]	0.693
**Electrocardiographic data**			
Right bundle branch block	27 (15.2%)	13 (14.6%)	1.000
Left bundle branch block	13 (7.3%)	8 (9.0%)	0.809
Atrioventricular block	27 (15.2%)	13 (14.6%)	1.000
**MDCT data**			
Annular area, cm^2	3.6 [3.5;4.4]	3.7 [3.5;4.5]	0.404
Annulus diameter, mm	23.9 [22.9;25.3]	23.9 [22.5;25.2]	0.487
LVOT, mm	23.2 [21.6;25.0]	23.5 [21.6;24.8]	0.880
STJ, mm	27.7 [25.9;29.8]	28.0 [26.5;29.9]	0.551
Aortic valve calcification, AU	2171 [1567;3117]	2488 [1585;3464]	0.429
Calcium density, AU/cm^2	558 [416;815]	644 [395;918]	0.494
Calcification in LVOT	14 (7.9%)	9 (10.1%)	0.700
Eccentric calcification	20 (11.2%)	10 (11.2%)	1.000

Abbreviations: PA = porcelain aorta, BMI = body mass index; eGFR = estimated glomerular filtration rate; LV = Left ventricle; AVA = aortic valve area; LVOT = left ventricular outflow tract; STJ = sinotubular junction. .

Table S2: Procedural outcomes and complications (matched population for classic PA)

Variable	**Non PA (2:1)** **n=178**	**Circular PA** **n=89**	**p Value**
**Procedural parameter**			
Procedural duration, min	45.00 [37.25;57.00]	47.00 [37.00;65.25]	0.623
Contrast agent, ml	93.50 [70.00;118.75]	90.00 [55.00;120.00]	0.607
Pre-dilatation, %	144 (80.90%)	72 (80.90%)	1.000
Post-dilatation, %	60 (34.68%)	28 (31.46%)	0.700
Protection device, %	4 (2.25%)	1 (1.12%)	0.668
Depth NCC, mm	5.95 [4.00;7.00]	6.00 [4.00;7.00]	0.237
Depth LCC, mm	6.00 [4.30;7.00]	6.00 [5.00;7.00]	0.269
Cover Index (annulus)	5.19 [3.21;7.20]	5.43 [3.28;7.23]	0.910
**Echocardiographic outcome**			
LV Ejection fraction, %	65.00 [56.00;65.00]	63.00 [53.00;65.00]	0.491
Mean gradient, mmHg	8.00 [6.00;10.50]	8.00 [6.00;11.00]	0.788
AVA, cm^2	1.70 [1.50;2.00]	1.72 [1.45;1.90]	0.658
iAVA, cm^2/m^2	0.96 [0.81;1.10]	0.94 [0.85;1.05]	0.703
**Procedural and clinical outcome**			
Technical success	159 (89.33%)	76 (85.39%)	0.464
Device success at 30 days	148 (83.15%)	72 (80.90%)	0.776
Early safety at 30 days	93 (52.25%)	43 (48.31%)	0.634
Inhospital death	2 (1.12%)	0 (0.00%)	0.554
Periprocedural death (inhospital and up to 30 days)	4 (2.25%)	2 (2.25%)	1.000
Relevant PVL (> mild/trace)	9 (5.06%)	4 (4.49%)	1.000
More than mild PPM	6 (4.29%)	4 (6.06%)	0.729
Conversion to sternotomy	1 (0.56%)	0 (0.00%)	1.000
Multiple valves (ViV)	5 (2.81%)	1 (1.12%)	0.667
Device embolization	6 (3.37%)	1 (1.12%)	0.430
Major vascular complication	14 (7.87%)	12 (13.48%)	0.215
Bleeding (type 2-4)	38 (21.35%)	22 (24.72%)	0.641
Major cardiac structural complication	1 (0.56%)	0 (0.00%)	1.000
All stroke	5 (2.81%)	4 (4.49%)	0.487
Neurologic dysfunction without CNS injury (TIA)	3 (1.69%)	0 (0.00%)	0.553
AKI (type 2-4)	9 (5.06%)	3 (3.37%)	0.756
New permanent pacemaker[1]	18 (11.18%)	12 (15.19%)	0.385

Abbreviations: PA = porcelain aorta; THV = transcatheter heart valve; LCC = left coronary cusp; NCC = non coronary cusp; AVA = aortic valve area; iAVA = indexed aortic valve area; PVL = paravalvular leak; CNS = central nervous system; ppm = prosthesis-patient mismatch; AKI = acute kidney injury.
[1]Excluded patients with pacemaker at baseline (n=27).

Table S3: Procedural outcomes and complications (entire population)

Variable	Non PA n=2518	Partial PA n=403	Circular PA n=89	p Value
**Procedural parameter**				
Procedural duration, min	45 [37;59]	50 [40;66]	47 [37;65]	<0.001
Contrast agent, ml	90 [60;115]	95 [70;120]	90 [55;120]	0.008
Pre-dilatation, %	1967 (78.12%)	344 (85.36%)	72 (80.90%)	0.004
Post-dilatation, %	821 (32.89%)	130 (32.42%)	28 (31.46%)	0.947
Protection device, %	63 (2.50%)	9 (2.23%)	1 (1.12%)	0.857
Depth NCC, mm	6.00 [4.00;6.50]	6.00 [4.00;7.00]	6.00 [4.00;7.00]	0.030
Depth LCC, mm	6.00 [4.00;7.00]	6.00 [4.80;7.00]	6.00 [5.00;7.00]	<0.001
Cover Index (annulus)	4.84 [2.83;7.01]	4.46 [2.39;6.37]	5.43 [3.28;7.23]	0.014
**Echocardiographic outcome**				
LV Ejection fraction, %	65 [56;65]	63 [55;65]	63 [53;65]	0.052
Mean gradient, mmHg	8.00 [6.00;11.00]	8.00 [6.00;11.00]	8.00 [6.00;11.00]	0.166
AVA, cm^2	1.70 [1.50;2.00]	1.80 [1.52;2.10]	1.72 [1.45;1.90]	0.045
iAVA, cm^2/m^2	0.93 [0.80;1.07]	0.94 [0.83;1.14]	0.94 [0.85;1.05]	0.119
**Procedural and clinical outcome**				
Technical success	2280 (90.55%)	354 (87.84%)	76 (85.39%)	0.081
Device success at 30 days	2131 (84.63%)	329 (81.64%)	72 (80.90%)	0.219
Early safety at 30 days	1057 (41.98%)	199 (49.38%)	43 (48.31%)	0.013
Inhospital death	52 (2.07%)	11 (2.73%)	0 (0.00%)	0.286
Periprocedural death (inhospital and up to 30 days)	70 (2.78%)	14 (3.47%)	2 (2.25%)	0.694
Relevant PVL (> mild/trace)	95 (3.78%)	16 (3.97%)	4 (4.49%)	0.820
More than mild PPM	74 (3.73%)	13 (4.64%)	4 (6.06%)	0.366
Conversion to sternotomy	25 (0.99%)	2 (0.50%)	0 (0.00%)	0.651
Multiple valves (ViV)	28 (1.11%)	4 (0.99%)	1 (1.12%)	1.000
Device migration / embolization	36 (1.43%)	7 (1.74%)	1 (1.12%)	0.876
Major vascular complication	200 (7.95%)	33 (8.19%)	12 (13.48%)	0.172
Bleeding (type 2-4)	443 (17.60%)	95 (23.57%)	22 (24.72%)	0.005
Major cardiac structural complication	43 (1.71%)	4 (0.99%)	0 (0.00%)	0.391
All stroke	79 (3.14%)	9 (2.23%)	4 (4.49%)	0.425
Neurologic dysfunction without CNS injury (TIA)	15 (0.60%)	6 (1.49%)	0 (0.00%)	0.140
AKI (type 2-4)	109 (4.33%)	12 (2.98%)	3 (3.37%)	0.482
New permanent pacemaker[1]	220 (9.84%)	40 11.30%)	12 (15.19%)	0.247

Abbreviation: PA = porcelain aorta; THV = transcatheter heart valve; LCC = left coronary cusp; NCC = non coronary cusp; AVA = aortic valve area; iAVA = indexed aortic valve area; PVL = paravalvular leak; CNS = central nervous system; ppm = prosthesis-patient mismatch; AKI = acute kidney injury.
[1]Excluded patients with pacemaker at baseline (n=342).

Table S4: Matching Algorithm

Variables used for propensity matching		
Clinical / ECG	CHA_2DS_2VASc	MDCT
EuroSCORE II	Atrial fibrillation	Cusp calcification (AU)
Previous PCI	Age	LVOT calcification
Diabetes	Gender	Eccentric AV calcification
COPD	Previous CAD	
Left branch bundle block	Previous stroke	
Right branch bundle block	PAD	
Creatinin clearance (eGFR)	aHT	

Abbreviations: AV = aortic valve; COPD = chronic obstructive pulmonary disease; CAD = coronary artery disease, MDCT = multidetector computed tomography; PCI = percutaneous coronary intervention, PAD = peripheral arterial disease, aHT = arterial hypertension.

Table S5: Technical Failure

Variable	**Non PA (2:1) n=984**	**PA n=492**	**p Value**
**Technical failure**	113 (11.5%)	62 (12.6%)	0.589
Death on table, %	3 (0.3%)	2 (0.4%)	1.000
Moderate paravalvular leakage, %	28 (2.9%)	18 (3.7%)	0.504
Major vascular complication	70 (7.1%)	45 (9.1%)	0.204
Major cardiac structural complication	14 (1.4%)	4 (0.8%)	0.450

Abbreviation: PA = porcelain aorta.
